# Neighbourhoods’ social, built, and natural environment characteristics and body mass index in Latin American cities

**DOI:** 10.1093/ije/dyaf047

**Published:** 2025-04-21

**Authors:** Santiago Rodríguez López, Ana V Diez Roux, Natalia Tumas, Kari Moore, Olga Lucía Sarmiento, Brisa N Sánchez, Carolina Pérez-Ferrer, Sandra Flores-Alvarado, Mónica Mazariegos, Usama Bilal, Mariana Lazo

**Affiliations:** Center of Research and Studies on Culture and Society, National and Technical Research Council and National University of Córdoba (CIECS, CONICET and UNC), Córdoba, Argentina; Department of Physiology, Faculty of Exact, Physical and Natural Sciences, National University of Córdoba, Córdoba, Argentina; Urban Health Collaborative, Dornsife School of Public Health, Drexel University, Philadelphia, PA, United States; Department of Epidemiology and Biostatistics, Dornsife School of Public Health, Drexel University, Philadelphia, PA, United States; Center of Research and Studies on Culture and Society, National and Technical Research Council and National University of Córdoba (CIECS, CONICET and UNC), Córdoba, Argentina; Faculty of Medical Sciences, National University of Córdoba, Córdoba, Argentina; Johns Hopkins University—Universitat Pompeu Fabra Public Policy (JHU-UPF PPC), Universitat Pompeu Fabra (UPF) - UPF Barcelona School of Management (UPF-BSM), Barcelona, Spain; Department of Epidemiology and Biostatistics, Dornsife School of Public Health, Drexel University, Philadelphia, PA, United States; School of Medicine, Universidad de Los Andes, Bogotá, Colombia; Department of Epidemiology and Biostatistics, Dornsife School of Public Health, Drexel University, Philadelphia, PA, United States; Center for Research in Population Health, National Institute of Public Health, Mexico; Program of Biostatistics, School of Public Health, University of Chile, Santiago, Chile; INCAP Research Center for the Prevention of Chronic Diseases (CIIPEC), Institute of Nutrition of Central America and Panama (INCAP), Guatemala City, Guatemala; Urban Health Collaborative, Dornsife School of Public Health, Drexel University, Philadelphia, PA, United States; Department of Epidemiology and Biostatistics, Dornsife School of Public Health, Drexel University, Philadelphia, PA, United States; Urban Health Collaborative, Dornsife School of Public Health, Drexel University, Philadelphia, PA, United States; Department of Community Health and Prevention, Dornsife School of Public Health, Drexel University, Philadelphia, PA, United States

**Keywords:** neighbourhood, education, intersection density, green space, street connectivity, Latin America

## Abstract

**Background:**

Features of neighbourhoods affect body mass index (BMI) but this has been poorly acknowledged within the highly heterogeneous and unequal contexts of Latin American cities. We evaluated associations between social, built, and natural environment characteristics of neighbourhoods with BMI, and investigated whether these associations were modified by individual socioeconomic position (SEP).

**Methods:**

We linked individual data (*n* = 43 968) from national health surveys to data on neighbourhoods (*n* = 3428) and cities (*n* = 165) in Argentina, Chile, Colombia, and Mexico. Linear mixed models were used to estimate associations between neighbourhood education, intersection density, and greenness with BMI, adjusting for individual- and city-level characteristics.

**Results:**

Associations between neighbourhood education and BMI varied by country, in both magnitude and direction. In Argentina and Chile, higher neighbourhood education was associated with lower BMI. This negative association was also observed among women in Colombia and Mexico, although it was weaker. Among men in Colombia and Mexico, however, the association was positive. Associations of neighbourhood intersection density and greenness with BMI were less robust. In general, we did not find strong evidence of effect modification by individual SEP.

**Conclusion:**

Neighbourhood education is associated with BMI beyond individual and city characteristics, although the associations are heterogenous across countries and by gender. Associations with built and natural features were less clear. Our results highlight the relevance of context-specific analysis for planning interventions that are aimed to reduce BMI and its unequal distribution in Latin American cities.

Key Messages

- We examined how neighbourhood features—social, built, and natural environments—are associated with body mass index (BMI) in 165 Latin American cities and whether these associations are modified by individual socioeconomic position (SEP) and gender.
- The association between neighbourhood education and BMI differs between countries and gender whereas the associations with intersection density and greenness and the effect modifications by individual SEP are less clear.
- Our findings suggest that neighbourhood characteristics in cities of Latin America appear to influence BMI differently from those in the Global North.

## Introduction

The growing burden of overweight and obesity since the 1980s is a major public health issue [1]. In 2015, it was estimated that high body mass index (BMI) contributed to 4 million deaths—7.1% of total deaths [2]—with significant variations across regions and countries. The temporal and spatial nature of this phenomenon points to the social origins of population-level increases in BMI [3]. Specifically, increases in BMI and the prevalence of overweight and obesity in low- and middle-income countries (LMICs) are often ascribed to changes in global trade patterns or increases in national income [4]. These changes are likely to affect people differently based on their place of residence and/or socioeconomic position (SEP) [4].

A large body of evidence, mainly from North America and Europe, describes associations between neighbourhood SEP and the nutritional status of individuals [5, 6]. Most of these studies have shown that individual and neighbourhood SEPs are independently and inversely associated with BMI. In addition, features of the built or natural environment in the place of residence have also been linked to BMI or obesity. For example, many studies that evaluated indicators of street connectivity or walkability such as intersection density at the neighbourhood level demonstrate an association with a higher likelihood of walking and physical activity [7] and also an inverse association with BMI or obesity [8]. Neighbourhood greenness has also been found to be inversely associated with overweight indicators [9]. Overall, longitudinal evidence suggests that long-term exposure to residential environments with greater resources to support physical activity is associated with a lower incidence of obesity-related outcomes [10].

Neighbourhood features may affect obesity and related outcomes independently of individual SEP through several mechanisms, including effects on health behaviours and stress-related processes [11], the availability of healthy foods [12], or physical-activity-promoting resources in neighbourhoods [13]. For example, low-SEP neighbourhoods likely have a lower number of exercise-promoting resources [14], creating barriers to engaging in physical activity [15]. In addition, the effect of neighbourhood features might differ according to individual-level SEP; for instance, a higher neighbourhood SEP may benefit cardiovascular health for those of lower, not higher, individual SEP [16].

Most studies on neighbourhood features and BMI have been conducted in high-income countries in the Global North and it is unclear whether these patterns of associations are also observed in other regions of the world. Latin America is one of the most urbanized and unequal regions in the world, with fast urban growth and inadequate urban planning [17], which result in large social inequalities. In addition, Latin America is one of the regions with the highest prevalence of adult obesity [18]. Providing knowledge on how disparities in BMI related to the neighbourhood’s social and built environment vary within and between Latin American cities is important for planning healthier and more equitable cities in the region. We expect to find different associations in Latin American cities compared with those of the Global North given the faster pace of urbanization and that neighbourhood environments are more heterogeneous. Particularly, and due to the social heterogeneity of Latin American cities, we expect to find stronger associations for characteristics of the social environment of neighbourhoods compared with other neighbourhood features.

Our study aims to (i) evaluate associations of neighbourhood social, built, and natural environment factors with individual-level BMI; and (ii) investigate whether these associations are modified by individual SEP. We hypothesize that (i) neighbourhood education, intersection density, and greenness are inversely associated with BMI, independently of individual and city characteristics, and (ii) these inverse associations of neighbourhood education, intersection density, and greenness with BMI are stronger among participants with lower individual education than those with higher education.

## Methods

### Data and sample

Data was compiled and harmonized by the SALURBAL project—an interdisciplinary, multinational, and collaborative initiative that is focused on characterizing the drivers of urban health and urban health inequalities across cities with a population of >100 000 inhabitants in 11 Latin American countries [19]. In our study, the people living in these cities represent the majority of the population in every country (ranging from 60% to 71%). We used harmonized data from Argentina, Chile, Colombia, and Mexico from (i) national representative health surveys linked to contextual data, (ii) aggregated socioeconomic and socio-demographic characteristics from a national population census, (iii) street network maps (OpenStreetMap), and (iv) remote sensing of green space and land cover (Finer Resolution Observation and Monitoring—Global Land Cover). Supplementary Table S1 provides the characteristics of the health surveys that were used to derive the sample at the individual level. Supplementary Table S2 summarizes the characteristics at the neighbourhood and city levels. Survey (and census) years were 2013 (2010) for Argentina, 2017 (2017) for Chile, 2007 (2005) for Colombia, and 2012 (2010) for Mexico.

As a proxy for the neighbourhoods, we used the administrative delimited units for census data collection. Neighbourhoods vary in population and areal size between countries (Supplementary Table S2), ranging from a median population of 1700 (Mexico) to 10 600 (Argentina). We focused on adults aged ≥18 years (except for Colombia, which only sampled participants aged 18–69 years) who participated in modules for which anthropometric measures were collected (*n* = 47 642). The final analytic sample included 43 968 participants from 165 cities and 3418 neighbourhoods (see Supplementary Figure S1 for further details). Included participants comprised a greater proportion of women and had slightly higher individual-level education and BMI compared with those who were excluded (Supplementary Table S3).

### Outcome

We used individual-level BMI [weight (kg)/height (m)^2^] as the outcome. For all countries, except for Argentina, weight and height were measured by following standard procedures. For Argentina, we used self-reported weight and height as physical measures were not part of the survey.

### Exposures

As a proxy for neighbourhood-level SEP, we used the proportion of the population aged ≥25 years who had completed primary education or above—obtained from a population census—with higher levels representing neighbourhoods with higher SEP. As the built environment indicator, we used intersection density to proxy street connectivity, defined as the number of intersections per km^2^ (nodes/km^2^). The higher the value of this metric, the greater the degree of connectivity, which would thus enable more direct travel between two points by using existing streets and facilitate active transportation. As the natural environment indicator, we used the proportion of green spaces in neighbourhoods that was obtained by dividing the total green-space area by the land area of the geographic unit. Green-space area and land area were net of water bodies and excluded limited no-observation areas in remote sensing images (due to cloud and shadow). Details on the measurement of each exposure are available elsewhere [19]. All continuous exposure variables were standardized by country with mean = 0 and SD = 1.

### Other variables

We obtained data on individual-level age, gender, and education (primary incomplete, primary complete, secondary complete, university complete) from the health surveys. We also linked city-level per-capita Gross Domestic Product [GDP, derived from modelled data in constant 2011 international United States dollars (USD)] and log-transformed city-level total population from population projections [20].

### Statistical analysis

We conducted the analysis in four steps. First, we described the distribution of individual, neighbourhood, and city characteristics by gender and country. Second, we estimated the variability in BMI between neighbourhoods and cities within countries by running a four-level pooled empty linear mixed model of individuals nested in neighbourhoods, cities, and countries, and calculated the percentage of the variance at each level. Third, to explore the associations between neighbourhood characteristics and BMI (Hypothesis 1), we ran a series of three-level random-intercept linear mixed models of individuals nested in neighbourhoods and cities, with a normally distributed random error at each level. All models were stratified by country and gender. Models 1a–c included one neighbourhood exposure at a time—(a) education, (b) intersection density, or (c) greenness—and were adjusted for age (linear), age-squared, per-capita city GDP (linear), and city total population (log-transformed). Models 2a–c had individual education added to Models 1a–c to test whether these associations held after adjusting for individual SEP. Model 3 had adjustments for the other neighbourhood exposures added to Models 2a–c (mutually adjusted model). Fourth, to test the effect of measure modification by individual SEP (Hypothesis 2), we added an interaction term between each main exposure in Models 2a–c and individual-level education, i.e. each interaction was tested in a separate model. Statistical analyses were performed by using Strata 15 and R.

## Results

Women have slightly higher age-adjusted BMI compared with men in all countries except Argentina (Table 1). Participants from Colombia have lower age-adjusted BMI. There are differences in the individual-level educational background across countries, with adults from Argentina, Chile, and Colombia having a higher proportion of participants with secondary or higher education compared with adults living in Mexico. Neighbourhoods in Colombia have the lowest mean neighbourhood education and percentage of greenness but the highest levels of intersection density. Colombia also has a lower mean city GDP whereas Argentina has the highest mean city population compared with other countries (Table 1).

**Table 1.** Individual, neighbourhood, and city characteristics by gender in Argentina, Chile, Colombia, and Mexico—the SALURBAL study

	**Overall (*n* = 43** **968)**		**Argentina (*n* = 14** **748)**		Chile (*n = *2950)		Colombia (*n* = 3554)		**Mexico (*n* = 22** **716)**	
	Men	Women	Men	Women	Men	Women	Men	Women	Men	Women
**Individual characteristics**										
BMI [mean (SE)]	27.27 (0.03)	27.59 (0.03)	27.19 (0.05)	25.89 (0.06)	28.23 (0.14)	29.13 (0.13)	24.70 (0.11)	25.38 (0.10)	27.60 (0.05)	28.79 (0.05)
Age-adjusted BMI^a^ [mean (SE)]	27.31 (0.04)	27.57 (0.03)	27.10 (0.06)	25.81 (0.06)	28.13 (0.14)	28.76 (0.12)	24.73 (0.12)	25.55 (0.11)	27.67 (0.05)	28.86 (0.05)
Age (years) [mean (SD)]	42.4 (17.2)	43.1 (17.2)	43.8 (17.2)	45.5 (18.4)	48.9 (18.8)	50.5 (18.5)	37.8 (13.8)	38.0 (13.2)	41.5 (17.2)	41.3 (16.2)
Educational level (%)										
University	13.9	15.3	14.7	20.5	15.0	11.2	8.9	8.1	9.7	7.1
Secondary	44.8	43.8	37.7	37.2	34.9	31.7	42.8	43.4	21.6	21.3
Primary	29.6	29.1	39.1	34.1	40.1	44.9	36.5	35.0	50.4	51.0
Less than primary	11.7	11.8	8.5	8.2	10.0	12.2	11.8	13.5	18.3	20.6
**Neighbourhood characteristics^b^^,c^**										
Percentage of population with complete primary education or above [mean (SD)]	88.3 (6.8)		89.3 (5.8)		90.4 (5.3)		80.1 (8.7)		88.6 (6.3)	
Intersection density nodes/km^2^ [mean (SD)]	129.9 (130.0)		88.4 (42.1)		184.8 (95.1)		213.1 (349.9)		136.7 (92.6)	
Percentage of green space [mean (SD)]	36.6 (30.1)		39.9 (27.1)		22.1 (22.6)		21.4 (19.1)		38.8 (34.4)	
**City characteristics^c^**										
GDP per capita (thousand USD) [mean (SD)]	18.9 (14.6)		20.1 (8.0)		23.0 (14.3)		10.6 (3.4)		18.9 (18.1)	
Total population (millions) [mean (SD)]	3.2 (5.7)		4.2 (6.3)		1.8 (2.6)		2.4 (2.8)		2.8 (5.7)	

SE, standard error.

a Age-adjusted BMI obtained from predicting BMI at the average sample age (42.8 years) from a linear regression model of BMI on linear and quadratic age; mean BMI (SD) in the overall sample 27.5 (5.3) kg/m^2^.

b The averages of neighbourhood characteristics are averaged over all neighbourhoods.

c Means and SD reported here were used in standardizing.

The mean BMI (SD) in the total sample was 27.5 (5.3) kg/m^2^. Overall, although most of the variability in BMI occurs at the individual level (90.3%), a modest proportion is accounted for by differences at the neighbourhood (1.1%), city (1.0%), and country (7.6%) levels (Supplementary Table S4). The neighbourhood variation in BMI is higher in Chile and Argentina (Supplementary Table S4).

**Figure 1.**
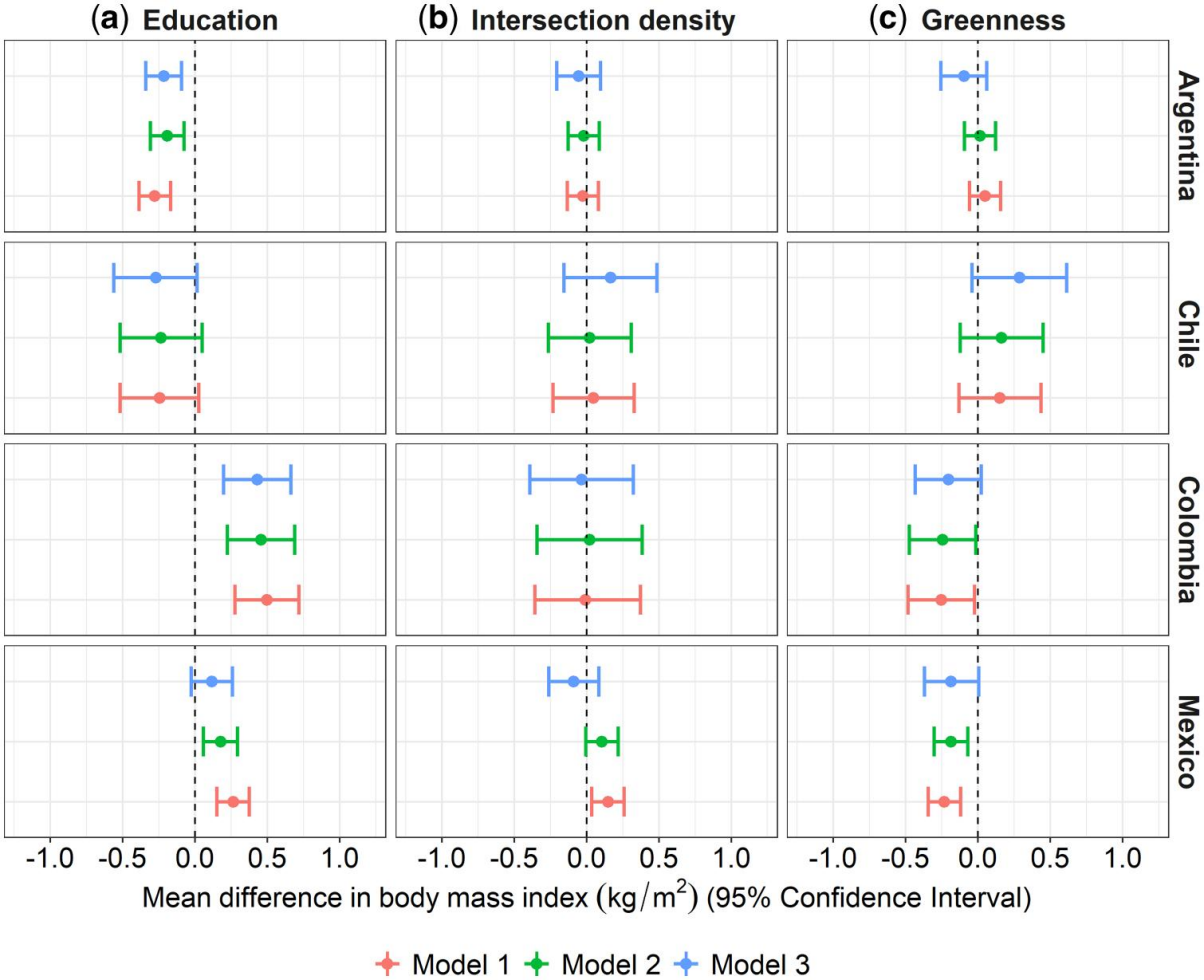
Adjusted association between neighbourhood (a) education (z-score), (b) intersection density (z-score), and (c) green space (z-score) and BMI (kg/m^2^) for men. Models 1 and 2: multilevel single-exposure models (each neighbourhood characteristic in a separate model) with random intercepts for neighbourhoods and cities; Model 3: multilevel multiple-exposure model (multiple exposures together). Model 1: analyses are adjusted by age (linear continuous), age-squared, city GDP per capita (z-score linear continuous), and city total population (z-score log-linear continuous); Model 2 adds individual education to Model 1; Model 3 adds the other neighbourhood exposures to Model 2. All continuous exposure variables are standardized by country with mean = 0 and SD = 1.

**Figure 2.**
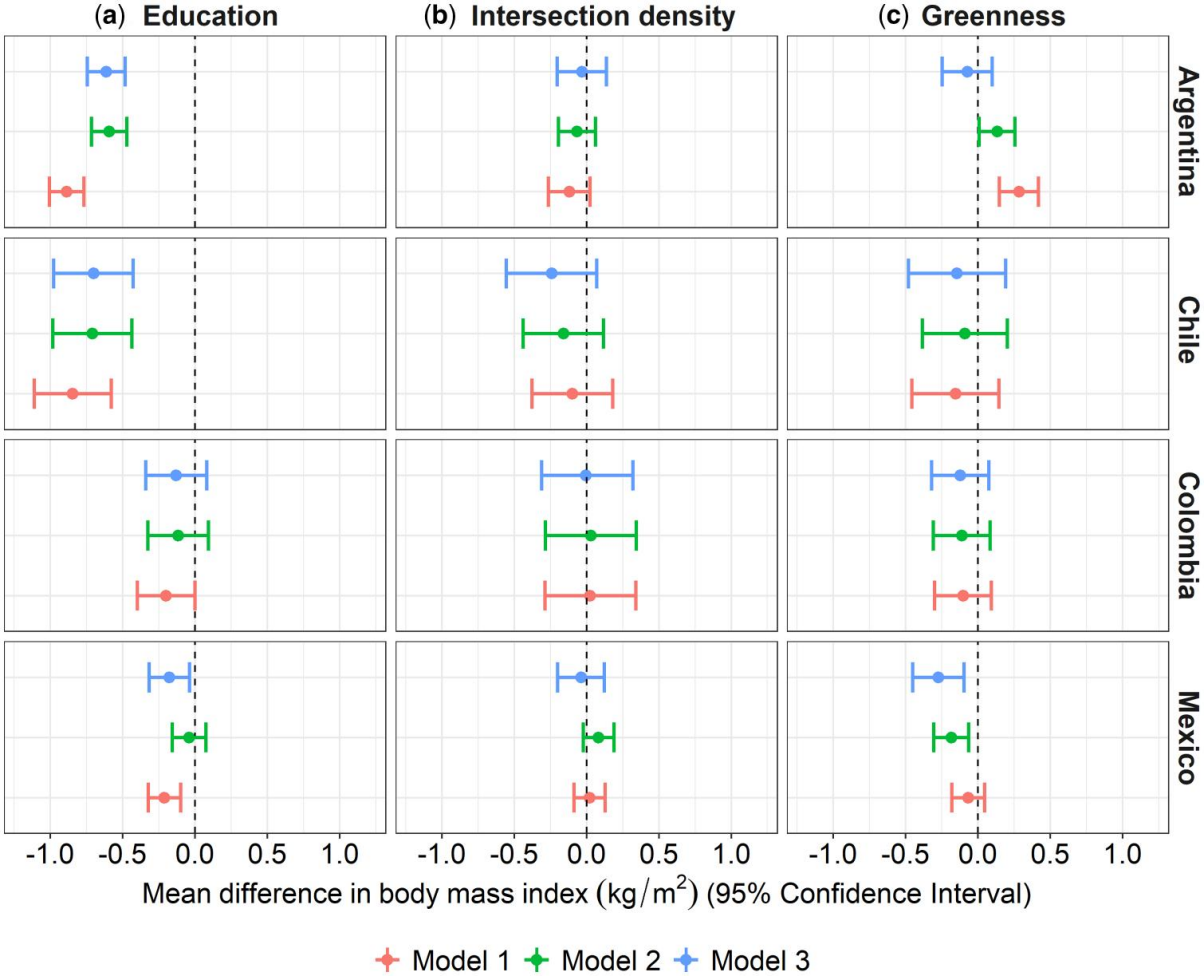
Adjusted association between neighbourhood (a) education (z-score), (b) intersection density (z-score), and (c) green space (z-score) and BMI (kg/m^2^) for women. Models 1 and 2: multilevel single-exposure models (each neighbourhood characteristic in a separate model) with random intercepts for neighbourhoods and cities; Model 3: multilevel multiple-exposure model (multiple exposures together). Model 1: analyses are adjusted by age (linear continuous), age-squared, city GDP per capita (z-score linear continuous), and city total population (z-score log-linear continuous); Model 2 adds individual education to Model 1; Model 3 adds the other neighbourhood exposures to Model 2. All continuous exposure variables are standardized by country with mean = 0 and SD = 1.

There are inverse associations between neighbourhood education and BMI in Argentina and Chile for both men and women, even after adjusting for individual and city characteristics (Models 1 and 2a, Figures 1 and 2). These associations are stronger among women compared with men, with a 1-SD higher unit in the percentage of the population with complete primary education or above associated with 0.593 (Confidence Interval (CI) –0.716, –0.470) and 0.709 (CI –0.984, –0.435) kg/m^2^ lower BMI in women versus 0.192 (CI –0.308, –0.075) and 0.235 (CI –0.519, 0.049) kg/m^2^ lower BMI in men from Argentina and Chile, respectively, after adjusting for individual-level education (Model 2a). These associations hold after adjusting for other neighbourhood exposures (Model 3a). In Colombia and Mexico, we also observe inverse associations between neighbourhood education and BMI among women but the associations are weaker. In contrast, among men living in Colombia and Mexico, we observe a pattern in the opposite direction, with a 1-SD higher unit in neighbourhood education being associated with 0.456 (CI 0.224, 0.689) and 0.177 (CI 0.058, 0.295) kg/m^2^ higher BMI in men from Colombia and Mexico, respectively (Model 2a; individual education model). In multiple-exposure models (Models 3), associations were scarcely attenuated (for men) and strengthened (for women) only in Mexico (see Supplementary Tables S5 and S6 for the complete estimates of the association analyses).

For intersection density (Models 2b and 3), associations with BMI were null among men and women in all countries. For neighbourhood greenness, after adjusting for individual education (Model 2c), associations with BMI among men and women were null in Chile, slightly positive in Argentina (women β 0.132; CI 0.009, 0.255), and negative in Mexico [1-SD higher unit in neighbourhood greenness associated with 0.187 (CI –0.304, –0.070) and 0.185 (CI –0.306, –0.064) lower BMI in men and women, respectively] and Colombia (although weaker). When adjusting for multiple exposures (Models 3), associations were attenuated to the null in Argentina and to a lesser extent in Mexico (Figures 1 and 2, and Supplementary Tables S5 and S6).

**Figure 3.**
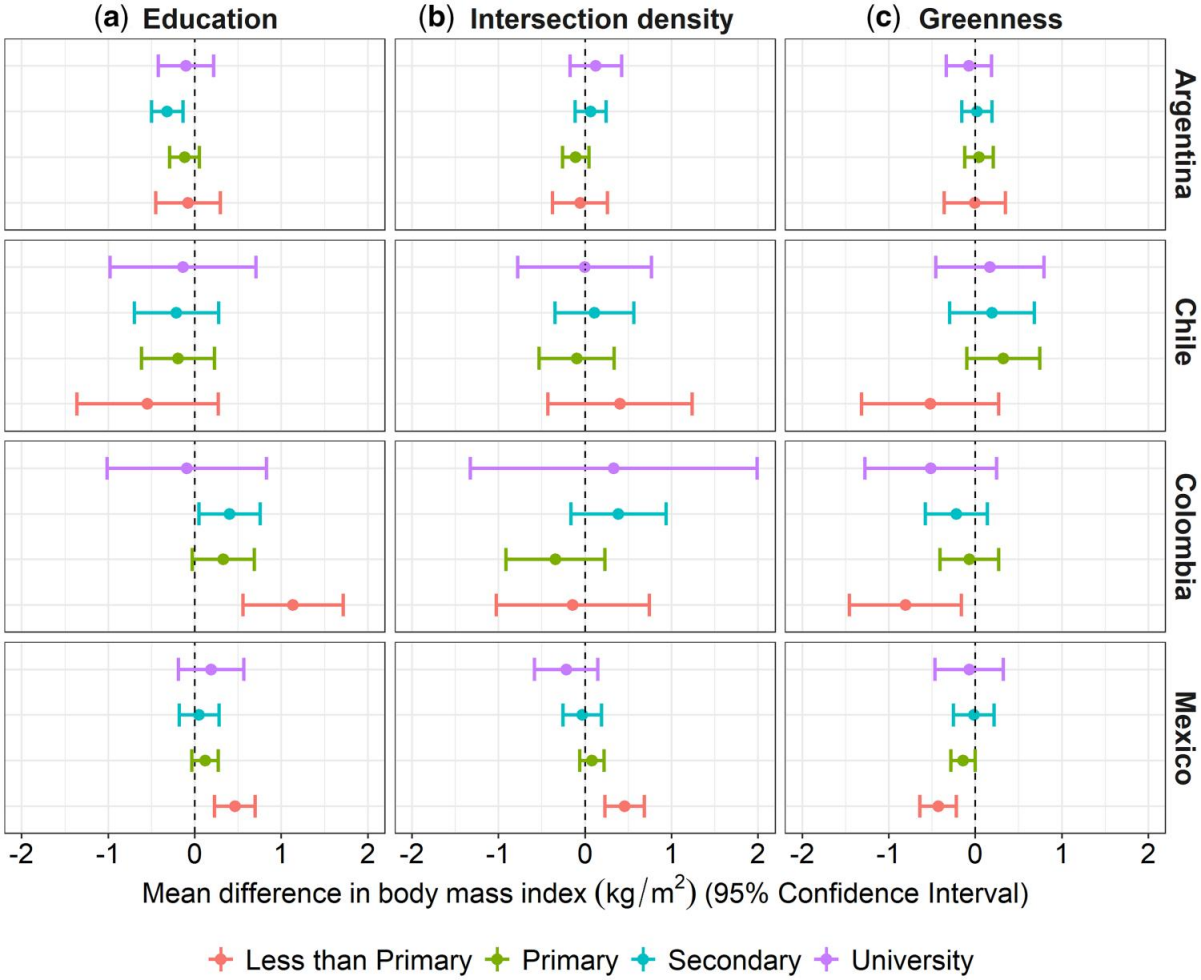
Mean differences (95% CI) in BMI per 1-SD higher value in the neighbourhood characteristic by levels of individual education in Argentina, Chile, Colombia, and Mexico (men). Adjusted multilevel single-exposure models (each neighbourhood characteristic in a separate model) including all main effects and the interaction between the neighbourhood characteristic (percentage of the population with complete primary education or above/intersection density/percentage of greenness) and individual education, and random intercepts for neighbourhood and cities. Models are adjusted by age (linear continuous), age-squared, city GDP per capita (z-score linear continuous), and city total population (z-score log-linear continuous). All continuous exposure variables are standardized by country with mean = 0 and SD = 1. Significant global *P*-value for interaction: percentage of population with complete primary education (Mexico; *P* < 0.05), intersection density (Mexico; *P* < 0.05), and percentage of green space (Mexico; *P* < 0.05).

**Figure 4.**
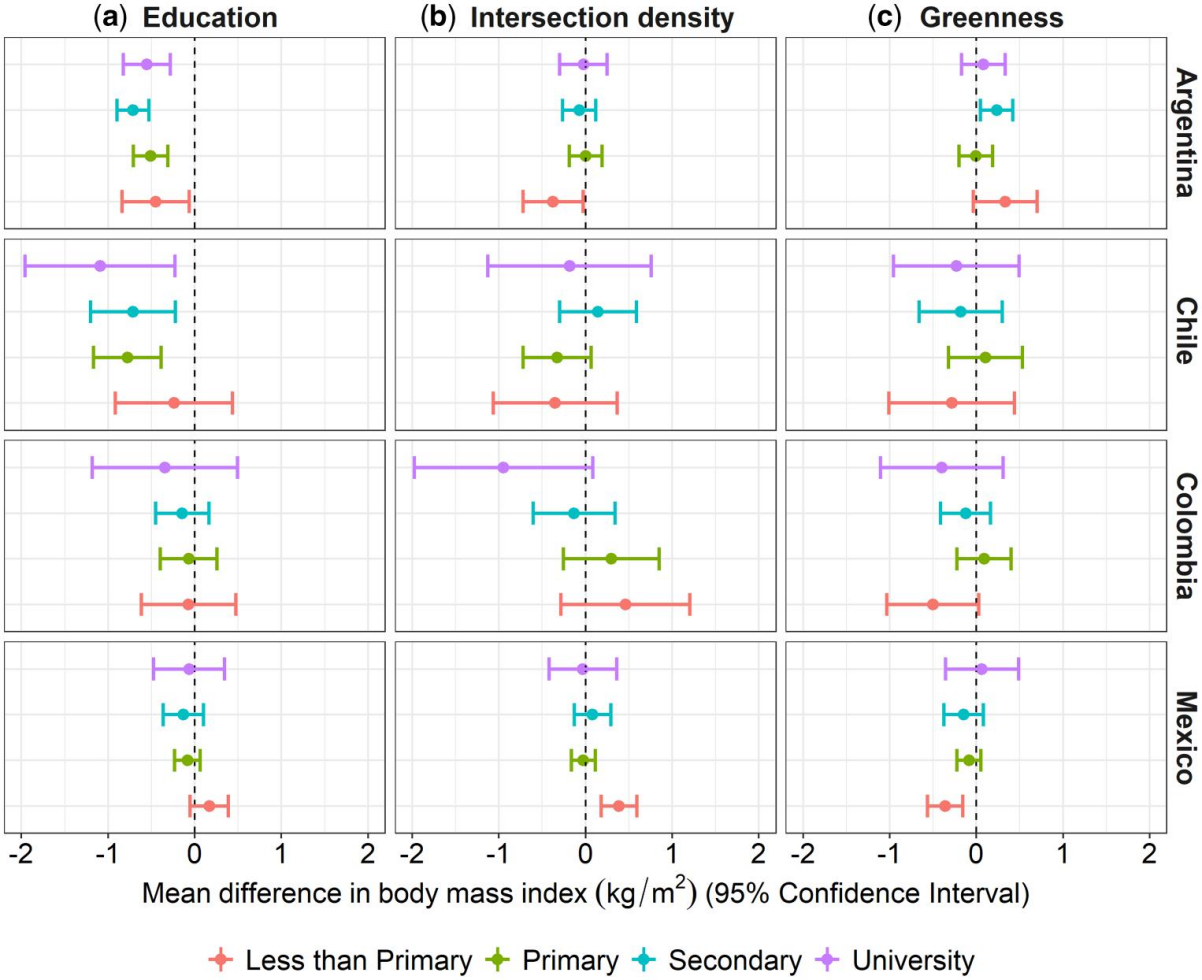
Mean differences (95% CI) in BMI per 1-SD higher value in the neighbourhood characteristic by levels of individual education in Argentina, Chile, Colombia, and Mexico (women). Adjusted multilevel single-exposure models (each neighbourhood characteristic in a separate model) including all main effects and the interaction between the neighbourhood characteristic (percentage of the population with complete primary education or above/intersection density/ percentage of greenness) and individual education, and random intercepts for neighbourhood and cities. Models are adjusted by age (linear continuous), age-squared, city GDP per capita (z-score linear continuous), and city total population (z-score log-linear continuous). All continuous exposure variables are standardized by country with mean = 0 and SD = 1. Significant global *P*-value for interaction: intersection density (Mexico; *P* < 0.05).

The positive association between neighbourhood education and BMI in men from Colombia and Mexico seems to be stronger among those with less than primary education compared with others (Figures 3 and 4, and Supplementary Table S7). Individuals with less than primary education living in neighbourhoods with higher intersection density had higher BMI in Mexico (men and women) but perhaps lower BMI in Argentina (women). For green space, although there were no clear differences across the educational categories in any country, the BMI of those who did not complete primary education in Mexico, Colombia, and Chile (men) seemed more negatively associated with greenness.

## Discussion

In this study, we explored the variability and characteristics of neighbourhoods associated with BMI in 43 968 individuals residing in 3418 neighbourhoods in 165 cities in four Latin American countries. We found inverse associations between neighbourhood education and BMI in Argentina and Chile, with higher neighbourhood-level education associated with lower BMI. In Colombia and Mexico, the pattern of associations was weaker (among women) or opposite (among men). Moreover, we found no consistent evidence for the association between BMI and neighbourhood intersection density or green space, except for a marginally inverse association with the latter in Mexico and Colombia. Finally, we did not find evidence of effect modification by individual SEP in the direction we hypothesized, except for the negative association between green space and BMI in Mexico and Colombia, where associations appear to be negative and stronger among individuals with the lowest SEP.

Effects of residential neighbourhood environments on health may vary across geographical space or individual SEPs [21], with differences in local contexts influencing how much neighbourhood characteristics matter for the health of local residents [22]. Previous work has found significant variation in BMI across neighbourhoods in high-income countries such as the USA [23] or England [22] but also a considerable range in neighbourhood variation in BMI in LMICs [21]. To the best of our knowledge, no prior research has quantified the neighbourhood variation in BMI in such a wide range of cities of different countries in Latin America. We found that the magnitude of the neighbourhood variation in BMI varied by country—highest in Chile and followed by Argentina—indicating the relative importance of neighbourhoods among the countries; i.e. in countries with greater neighbourhood variation, BMI may be more strongly influenced by local conditions than in countries with less variation attributed to neighbourhoods [21].

Our study showed interesting neighbourhood contextual education patterns. We hypothesized that neighbourhood education would be inversely associated with BMI, independently of individual and city characteristics. Although this was true in Argentina and Chile for both men and women, in Colombia and Mexico, this association was weaker among women and was actually the opposite among men. Our findings for Argentina and Chile are consistent with large amounts of evidence—mostly but not limited to high-income countries [6]—reporting lower BMI in neighbourhoods with higher SEP [5]. Moreover, we found gender differences in the strength of the associations: the inverse associations in Argentina and Chile were stronger among women whereas the direct associations in Colombia and Mexico were stronger among men. Differences in the neighbourhood SEP–BMI associations among countries could be because Argentina and Chile are in a more advanced stage of epidemiological and nutrition transitions compared with Colombia and Mexico. Our findings for Colombia and Mexico are aligned with recent research in Latin America describing a positive association between educational background and obesity for men living in cities with lower levels of development [24]. For Argentina, our results align with others that describe inverse associations between neighbourhood education and obesity in this country [25].

With some exceptions [26], considerable evidence [8] from high-income countries has found associations between greater neighbourhood walkability (commonly using intersection density as a proxy) and lower BMI, even including a study in Latin America [27]. Our null findings could be related to the unique features resulting from the rapid urbanization of Latin America, where higher intersection density does not necessarily indicate pedestrian-friendly pathways, and engagement in physical activity may even be limited by concerns of safety and violence [28].

We also tested the hypothesis that individuals living in greener neighbourhoods have lower BMI, finding mostly null results. Most of the literature from high-income countries such as the USA [29], UK [30], Australia [31], or China [9] described inverse associations between neighbourhood green space and BMI. Our null findings, with the exception of an inverse association in Mexico and less clearly in Colombia, suggest that the association of green spaces with BMI may not be universal; e.g. safety concerns in green spaces may influence their use [32].

Neighbourhood effects on BMI may also differ by individual SEP. We hypothesized that the inverse associations between characteristics of neighbourhoods and BMI would be stronger among participants with lower individual SEP than those with higher SEP. This seems to be true in Mexico and Colombia, where the negative associations between green space and BMI appear to be somehow stronger among individuals with the lowest SEP. In contrast, we found that most of the associations between neighbourhood characteristics and BMI did not differ substantially across individual education.

There are a number of important strengths of our work. To our knowledge, no prior study in Latin America has examined the importance of neighbourhood features on a large urban scale or analysed the interplay between proxies of the social, built, and natural environment and individual BMI. We included data from 4 countries, 165 cities, 43 968 individuals, and linked datasets containing harmonized, individual-level, neighbourhood, and city indicators. A limitation of our study is the cross-sectional nature of the data, which prevented us from deriving any type of causality from our study, and the possibility of residual confounding. Another limitation is that the definition of ‘neighbourhoods’ varied somewhat across countries and the characteristics of neighbourhoods were retrieved from different data sources that were not aligned with the years for which health survey data were obtained for each country. Therefore, we had to assume that neighbourhood characteristics were stable across the years examined (however, the maximum window of time between surveys and census was only 3 years). The social, built, and natural environments are much broader and more complex than the proxy variables that were employed in this study could reflect. Indeed, it is likely that intersection density per se might not be a good proxy for neighbourhood walkability in Latin America. We did not include data on neighbourhood infrastructure such as street lighting, security, etc., which might also be important determinants of how walkable the neighbourhoods in our study are. The use of self-reported weight and height in Argentina is also a limitation, which likely introduces measurement error and could be affected by access to care that may differ by SEP.

## Conclusions

In summary, our study provided the largest characterization of the links between social, built, and natural environment residential characteristics and BMI in cities of Latin America, suggesting that BMI is potentially and differently conditioned by neighbourhood features, beyond individual and city characteristics. Our findings suggest that neighbourhood characteristics in Latin America appear to influence BMI differently from those in the Global North, reinforcing the need for the generation of further evidence on residential associations in these settings. Interventions to reduce BMI and its unequal distribution should primarily encompass improving the neighbourhood social environment as well as targeted interventions in the most disadvantaged groups.

## Ethics approval

The SALURBAL study protocol was approved by the Drexel University Institutional Review Board with ID #1612005035 and by appropriate site-specific Institutional Review Boards.

## Supplementary Material

dyaf047_Supplementary_Data

## Data Availability

Some of the SALURBAL data in this study are available to download from the SALURBAL Portal: https://data.lacurbanhealth.org/data. Requests for the harmonized dataset can be obtained by contacting the SALURBAL project at salurbal.data@drexel.edu and after completing a data-use agreement. Requests are reviewed by the Data Methods Core and Publications & Presentations Committee on a monthly basis. To learn more about SALURBAL’s dataset, visit https://drexel.edu/lac/ or contact the project at salurbal@drexel.edu.
