# Supplementary material for: Neighbourhoods’ social, built, and natural environment characteristics and body mass index in Latin American cities
Source: Int J Epidemiol. 2025 Apr 21;54(3):dyaf047. doi: 10.1093/ije/dyaf047 (PMC12011360; doi:10.1093/ije/dyaf047)
Supplement: dyaf047_Supplementary_Data [file dyaf047_supplementary_data.pdf]

## Supplementary data

Table of contents

**Table S1. Health Survey data description including sample characteristics, sampling strategy, and representation ........ 2**

**Figure S1. Flow chart describing the sample selection, involving 165 cities and 3,418 neighbourhoods from four Latin American countries (Argentina, Chile, Colombia, and Mexico) ........ 3**

**Table S2. Study characteristics by country........4**

**Table S3. Comparison between included and excluded participants ........ 5**

**Table S4. Variance components for individual body mass index (BMI) in four Latin American countries (Argentina, Chile, Colombia, and Mexico) ........ 6**

**Table S5. Adjusted mean differences (95% CI) in body mass index (BMI) by neighbourhood characteristics in men ........ 7**

**Table S6. Adjusted mean differences (95% CI) in body mass index (BMI) by neighbourhood characteristics in women ........8**

**Table S7. Mean differences (95% CI) in body mass index (BMI) per 1 standard deviation (SD) higher value in the neighbourhood characteristic by levels of individual education in Argentina, Chile, Colombia and Mexico........ 9**

**Table S1.** Health survey data description including sample characteristics, sampling strategy, and representation.

| Health survey, Year, Country | Sample characteristics | Sample strategy | Geographic coverage | Representation |
|---|---|---|---|---|
| Encuesta Nacional de Factores de Riesgo, ENFR (National Risk Factors Survey) [2013], Argentina. | Age: ≥18 years; Total n: 32,365; n in SALURBAL: 21,451; n in this study: 14,748 | Multistage [Aglomerado censal; área (groups of radio censales); household; person 18 years or older]. Stratified [population size; education level of head of household]. | Localities with over 5,000 inhabitants | National, 6 regions, 23 provinces, Ciudad Autónoma de Buenos Aires, and 8 metropolitan areas >500,000 population |
| Encuesta Nacional de Salud, ENS (National Health Survey) [2017], Chile. | Age: ≥ 15 years; Total n: 6,233; n in SALURBAL: 3,805; n in this study: 2,950 | Multistage [Comunas; Segments within comunas; household; person 15 years or older]. | National | National, Regions (15), urban/rural |
| Encuesta Nacional de Salud, ENS (National Health Survey) [2007], Colombia. | Age: 0 – 69 years; Total n: 102,677 (41,281 adults 18-69 years); n in SALURBAL: 43,182 (18,783 adults 18-69 years completed module 2 or module 4); n in this study: 3,554 | Multistage [Municipalities or combination of municipalities if small; Manzanas; household; person adults 18-69 and all children 17 and under]. | National | Region, department, sub-region, urban area of municipal capitals, urban/rural, by poverty level |
| Encuesta Nacional de Salud y Nutrición, ENSANUT (National Survey for Health and Nutrition) [2012], Mexico. | Age: all ages; Total n: 46,277 adults ≥18 years; n in SALURBAL: 26,335 adults 18+ years; n in this study: 22,716 | Multistage [Área Geostadística Básica (AGEB); Manzana (urban) or pseudo-manzanas within localidades (rural); Households; 1 person within each of the groups (0-4 years, 5-9 years, 10-19 years, 20 years and older, recent medical service user)]. | National | National, state, metropolitan areas, urban/rural, high/low SES |

ENFR, Encuesta Nacional de Factores de Riesgo; ENS, Encuesta Nacional de Salud; ENSANUT, Encuesta Nacional de Salud y Nutrición; SALURBAL, Salud Urbana en América Latina; AGEB, Área Geostadística Básica; SES, socioeconomic status.

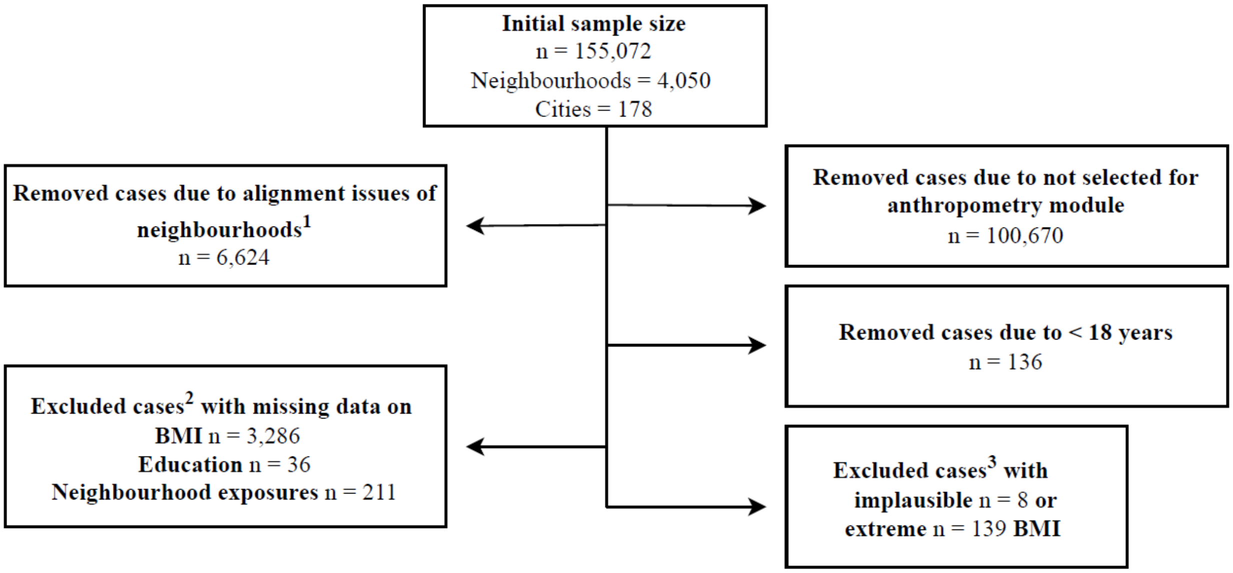


**Figure S1:** Flow chart describing the sample selection, involving 165 cities and 3,418 neighbourhoods from four Latin American countries (Argentina, Chile, Colombia, and Mexico).

[1]We excluded 426 neighbourhoods (including 6,624 survey respondents) for which the spatial boundaries used for census and built environment data collection differed.
[2]We excluded participants with missing data on BMI (n = 3,286), education (n = 36), or neighbourhood characteristics (n = 211).
[3]We also excluded 8 observations with implausible BMI and 139 observations of weight or height that deviate 4 SD below or above the sample average (BMI < 5.42 or > 49.67 kg/m$^2$) (see Yang & Hutcheon, 2016).
Yang S, Hutcheon JA. Identifying outliers and implausible values in growth trajectory data. *Ann Epidemiol* 2016; **26**: 77–80.
BMI, body mass index; SD, standard deviation

**Table S2.** Study sample characteristics at the neighbourhood and city level by country.

| Study characteristics | Argentina | Chile | Colombia | Mexico |
|---|---|---|---|---|
| Health survey, year | Encuesta Nacional de Factores de Riesgo, 2013 | Encuesta Nacional de Salud, 2017 | Encuesta Nacional de Salud, 2007 | Encuesta Nacional de Salud y Nutrición, 2012 |
| Cities, n (%) | 22 (13.3) | 21 (12.7) | 31 (18.8) | 91 (55.2) |
| Neighbourhood, n (%) | 1,000 (29.2) | 718 (21.1) | 783 (22.9) | 917 (26.8) |
| Participants, n (%) | 14,748 (33.6) | 2,950 (6.7) | 3,554 (8.0) | 22,716 (51.7) |
| Participants by neighbourhood, median ($25^{th}$ percentile, $75^{th}$ percentile) | 26 (13-42) | 5 (4-7) | 6 (4-9) | 29 (24-35) |
| Census, year | 2010 | 2017 | 2005 | 2010 |
| Population by neighbourhood (thousands)†, median ($25^{th}$ percentile, $75^{th}$ percentile) | 10.6 (7.3, 15.4) | 3.3 (2.4, 4.3) | 5.1 (2.2, 10.4) | 1.8 (.5, 3.1) |
| Size by neighbourhood †, median $km^2$ ($25^{th}$ percentile, $75^{th}$ percentile) | 2.3 (1.2, 8.1) | .37 (0.23, 0.65) | .41 (.21, .78) | .30 (.15, .52) |
| Neighbourhood denomination | Fracción censal | Zona censal | Sector urbano | AGEB |

†Calculated based on all the neighbourhoods of the SALURBAL project for Argentina (n = 2,409), Chile (n = 3,533), Colombia (n = 3,247), and Mexico (n=32,927), but not on the study sample. These were the countries for which we had social, built, and natural environment data at the neighbourhood scale. AGEB: Área Geoestadística Básica.

**Table S3.** Comparison between included and excluded participants.

| Individual characteristics | Excluded participants (n = 3,674) | Included participants (n = 43,968) | p-value |
|---|---|---|---|
| BMI†, mean (SD) | 28.5 (5.0) | 27.4 (5.3) | < .05 |
| Gender, % women | 45.8 | 57.5 | < .001 |
| Age (years), mean (SD) | 43.1 (19.6) | 42.8 (17.2) | .334 |
| Educational level, % | | | |
| University | 8.7 | 11.8 | < .001 |
| Secondary | 25.7 | 29.3 | |
| Primary | 45.8 | 44.2 | |
| Less than Primary | 19.8 | 14.7 | |

†Does not include participants excluded by implausible values nor those with extreme BMI (4 standard deviations (SD) below or above the sample average); p-values test the null hypothesis that there is no difference in the proportion ($\chi^2$) or mean (t-test/ANOVA) of the characteristic between excluded and included participants. BMI, body mass index; SD, standard deviation.

**Table S4.** Variance components for individual body mass index (BMI) in four Latin American countries (Argentina, Chile, Colombia, and Mexico).

| Random parameters | Variance (SE) | Proportion of variance at each level |
|---|---|---|
| ***Overall variation**** | | |
| Country (n = 4) | 2.214 (1.581) | 7.6 |
| City (n = 165) | .279 (.052) | 1.0 |
| Neighbourhood (n = 3,428) | .313 (.051) | 1.1 |
| Individual (n = 43,968) | 26.397 (.182) | 90.3 |
| ***Country-specific variation†*** | | |
| **Argentina** | | |
| City (n = 22) | .059 (.042) | .2 |
| Neighbourhood (n = 1,000) | .467 (.092) | 2.0 |
| Individual (n = 14,748) | 23.332 (.278) | 97.8 |
| **Chile** | | |
| City (n = 21) | .159 (.132) | .6 |
| Neighbourhood (n = 718) | 1.235 (.446) | 4.2 |
| Individual (n = 2,950) | 27.594 (.806) | 95.2 |
| **Colombia** | | |
| City (n = 31) | .154 (.106) | .7 |
| Neighbourhood (n = 783) | .191 (.232 | .8 |
| Individual (n = 3,554) | 21.154 (.543) | 98.5 |
| **Mexico** | | |
| City (n = 91) | .380 (.083) | 1.3 |
| Neighbourhood (n = 917) | .232 (.065) | .8 |
| Individual (n = 22,716) | 28.941 (.276) | 97.9 |

Outcome: individual BMI; *Four-level linear empty model with random intercepts for neighbourhoods, cities, and countries; † Country-specific three-level linear empty model with random intercepts for neighbourhoods and cities. BMI, body mass index; SE, standard error.

**Table S5.** Adjusted mean differences (95% CI) in body mass index (BMI) by neighbourhood characteristics in men.

| Neighbourhood characteristics | Mean differences in BMI (95% CI) | | |
|---|---|---|---|
| | Single-exposure models | | Multiple-exposure models |
| | Models 1a-c | Models 2a-c | Model 3 |
| **Argentina (n = 6,586, neighbourhoods = 942, cities = 22)** | | | |
| % of population with complete primary education or above, z-score | -.278 (-.386, -.169) | -.192 (-.308, -.075) | -.215 (-.339, -.092) |
| Intersection density, z-score | -.026 (-.134, .083) | -.019 (-.126, .088) | -.055 (-.206, .097) |
| % of greenspace, z-score | .048 (-.060, .156) | .013 (-.095, .120) | -.098 (-.258, .060) |
| **Chile (n = 1,095, neighbourhoods = 565, cities = 21)** | | | |
| % of population with complete primary education or above, z-score | -.245 (-.517, .025) | -.235 (-.519, .049) | -.272 (-.560, .015) |
| Intersection density, z-score | .049 (-.232, .331) | .022 (-.263, .308) | .166 (-.155, .487) |
| % of greenspace, z-score | .151 (-.132, .436) | .163 (-.123, .449) | .286 (-.042, .614) |
| **Colombia (n = 1,420, neighbourhoods = 606, cities = 31)** | | | |
| % of population with complete primary education or above, z-score | .496 (.275, .718) | .456 (.224, .689) | .431 (.198, .664) |
| Intersection density, z-score | -.009 (-.357, .374) | .022 (-.341, .386) | -.033 (-.391, .325) |
| % of greenspace, z-score | -.254 (-.483, -.024) | -.246 (-.475, -.016) | -.205 (-.434, .023) |
| **Mexico (n = 9,596, neighbourhoods = 914, cities = 91)** | | | |
| % of population with complete primary education or above, z-score | .264 (.151, .376) | .177 (.058, .295) | .115 (-.027, .258) |
| Intersection density, z-score | .148 (.036, .260) | .107 (-.005, .219) | -.088 (-.262, .086) |
| % of greenspace, z-score | -.232 (-.344, -.120) | -.187 (-.304, -.070) | -.188 (-.371, .005) |

Multilevel single- (each neighbourhood characteristic in a separate model) and multiple-exposure models with random intercepts for neighbourhoods and cities. Models 1a-c are adjusted by age, age-squared, city GDP per capita and city total population (log); Models 2a-c add individual education to models 1a-c; Model 3 adds neighbourhood exposures to models 2a-c. All continuous exposure variables are standardized by country with mean=0 and SD=1. BMI, body mass index; GDP, gross domestic product; SD, standard deviation.

**Table S6.** Adjusted mean differences (95% CI) in body mass index (BMI) by neighbourhood characteristics in women.

| Neighbourhood characteristics | Mean differences in BMI (95% CI) | | |
|---|---|---|---|
| | Single-exposure models | | Multiple-exposure models |
| | Models 1a-c | Models 2a-c | Model 3 |
| **Argentina (n = 8,162; neighbourhoods = 973; cities = 22)** | | | |
| % of population with complete primary education or above, z-score | -.887 (-1.007, -.767) | -.593 (-.716, -.470) | -.613 (-.744, -.483) |
| Intersection density, z-score | -.119 (-.263, .024) | -.067 (-.194, .061) | -.031 (-.202, .139) |
| % of greenspace, z-score | .283 (.148, .417) | .132 (.009, .255) | -.074 (-.248, .099) |
| **Chile (n = 1,855, neighbourhoods = 654, cities = 21)** | | | |
| % of population with complete primary education or above, z-score | -.846 (-1.112, -.579) | -.709 (-.984, -.435) | -.702 (-.977, -.428) |
| Intersection density, z-score | -.098 (-.377, .180) | -.160 (-.437, .117) | -.241 (-.554, .071) |
| % of greenspace, z-score | -.156 (-.457, .144) | -.091 (-.385, .204) | -.145 (-.480, .190) |
| **Colombia (n = 2,134, neighbourhoods = 703, cities = 31)** | | | |
| % of population with complete primary education or above, z-score | -.201 (-.400, -.001) | -.117 (-.327, .093) | -.130 (-.341, .080) |
| Intersection density, z-score | .025 (-.288, .340) | .030 (-.284, .344) | -.005 (-.311, .321) |
| % of greenspace, z-score | -.103 (-.299, .093) | -.112 (-.308, .084) | -.124 (-.322, .074) |
| **Mexico (n = 13,120, neighbourhoods = 914, cities = 91)** | | | |
| % of population with complete primary education or above, z-score | -.212 (-.324, -.099) | -.041 (-.158, .076) | -.177 (-.316, -.038) |
| Intersection density, z-score | .022 (-.085, .130) | .083 (-.023, .189) | -.038 (-.200, .124) |
| % of greenspace, z-score | -.068 (-.180, .045) | -.185 (-.306, -.064) | -.275 (-.451, -.098) |

Multilevel single- (each neighbourhood characteristic in a separate model) and multiple-exposure models with random intercepts for neighbourhoods and cities. Models 1a-c are adjusted by age, age-squared, city GDP per capita and city total population (log); Models 2a-c add individual education to model 1a-c; Model 3 adds neighbourhood exposures to models 2a-c. All continuous exposure variables are standardized by country with mean=0 and SD=1. BMI, body mass index; GDP, gross domestic product; SD, standard deviation.

**Table S7.** Mean differences (95% CI) in body mass index (BMI) per 1 standard deviation (SD) higher value in the neighbourhood characteristic by levels of individual education in Argentina, Chile, Colombia and Mexico.

| | **Mean differences in BMI (95% CI) by 1 SD higher value of the neighbourhood characteristic** | | | | |
|---|---|---|---|---|---|
| | **Individual education** | | | | |
| **Neighbourhood characteristics** | **Less than Primary** | **Primary** | **Secondary** | **University** | **Global P-value for interaction** |
| ***Men*** | | | | | |
| **Argentina (n=6,586)** | | | | | |
| % of population with complete primary education | -.079 (-.453, .295) | -.118 (-.293, .055) | -.318 (-.501, -.135) | -.102 (-.422, .218) | .349 |
| Intersection density | -.057 (-.374, .259) | -.108 (-.261, .045) | .063 (-.115, .242) | .124 (-.173, .422) | .364 |
| % of greenness | -.007 (-.361, .347) | .043 (-.120, .207) | .018 (-.155, .192) | -.074 (-.337, .187) | .897 |
| **Chile (n=1,095)** | | | | | |
| % of population with complete primary education | -.546 (-1.362, .270) | -.194 (-.615, .226) | -.213 (-.700, .274) | -.135 (-.978, .708) | .882 |
| Intersection density | .402 (-.432, 1.236) | -.097 (-.533, .337) | .108 (-.345, .562) | -.004 (-.777, .767) | .744 |
| % of greenness | -.522 (-1.316, .272) | .322 (-.098, .743) | .192 (-.298, .683) | .168 (-.457, .793) | .318 |
| **Colombia (n=1,420)** | | | | | |
| % of population with complete primary education | 1.136 (.558, 1.714) | .329 (-.027, .686) | .403 (.050, .756) | -.093 (-1.014, .827) | .062 |
| Intersection density | -.142 (-1.027, .741) | -.341 (-.913, .231) | .384 (-.162, .935) | .332 (-1.325, 1.989) | .309 |
| % of greenness | -.807 (-1.455, -.159) | -.069 (-.409, .271) | -.220 (-.578, .137) | -.514 (-1.277, .248) | .208 |
| **Mexico (n=9,596)** | | | | | |
| % of population with complete primary education | .463 (.230, .696) | .119 (-.032, .271) | .050 (-.181, .281) | .191 (-.187, .569) | .040 |
| Intersection density | .457 (.228, .686) | .079 (-.063, .222) | -.031 (-.255, .193) | -.217 (-.583, .148) | .027 |
| % of greenness | -.429 (-.640, -.218) | -.141 (-.283, .001) | -.018 (-.253, .216) | -.071 (-.466, .322) | .039 |

| | | | | | |
|---|---|---|---|---|---|
| ***Women*** | | | | | |
| **Argentina (n=8,162)** | | | | | |
| % of population with complete primary education | -.450 (-.839, -.061) | -.509 (-.707, -.311) | -.714 (-.899, -.528) | -.554 (-.826, -.283) | .381 |
| Intersection density | -.374 (-.722, -.025) | .003 (-.188, .193) | -.072 (-.266, .120) | -.024 (-.298, .249) | .281 |
| % of greenness | .332 (-.036, .701) | -.005 (-.201, .189) | .234 (.049, .419) | .081 (-.169, .331) | .188 |
| **Chile (n=1,855)** | | | | | |
| % of population with complete primary education | -.240 (-.916, .434) | -.778 (-1.168, -.388) | -.714 (-1.203, -.225) | -1.092 (-1.957, -.227) | .431 |
| Intersection density | -.350 (-1.067, .367) | -.326 (-.720, .067) | .143 (-.299, .587) | -.182 (-1.126, .760) | .405 |
| % of greenness | -.284 (-1.009, .441) | .103 (-.323, .531) | -.179 (-.660, .301) | -.230 (-.956, .495) | .687 |
| **Colombia (n=2,134)** | | | | | |
| % of population with complete primary education | -.071 (-.617, .474) | -.068 (-.396, .259) | -.145 (-.453, .163) | -.343 (-1.181, .494) | .933 |
| Intersection density | .461 (-.282, 1.206) | .297 (-.253, .848) | -.133 (-.606, .339) | -.946 (-1.975, .082) | .103 |
| % of greenness | -.502 (-1.032, .027) | .089 (-.222, .402) | -.124 (-.411, .163) | -.397 (-1.105, .310) | .219 |
| **Mexico (n=13,120)** | | | | | |
| % of population with complete primary education | .167 (-.053, .387) | -.085 (-.233, .062) | -.132 (-.364, .100) | -.064 (-.474, .345) | .182 |
| Intersection density | .386 (.179, .594) | -.025 (-.162, .112) | .081 (-.128, .291) | -.030 (-.422, .360) | .007 |
| % of greenness | -.360 (-.562, -.158) | -.086 (-.226, .053) | -.147 (-.375, .080) | .064 (-.357, .487) | .087 |

Adjusted multilevel single-exposure models (each neighbourhood characteristic in a separate model) including all main effects and the interaction between the neighbourhood characteristic (% of the population with complete primary education or above / Intersection density / % of greenness) and individual education, and random intercepts for neighbourhood and cities. Models are adjusted by age (linear continuous), age-squared, city GDP per capita (z-score linear continuous), and city total population (z-score log-linear continuous). All continuous exposure variables are standardized by country with mean=0 and SD=1. BMI, body mass index; GDP, gross domestic product; SD, standard deviation.
